# Ten‐step method of high‐dose LDR ^125^I brachytherapy for intermediate‐risk prostate cancer

**DOI:** 10.1002/acm2.13224

**Published:** 2021-05-03

**Authors:** Keisei Okamoto

**Affiliations:** ^1^ Department of Brachytherapy for Prostate Cancer Shiga University of Medical Science Shiga Japan

**Keywords:** low dose rate (LDR) brachytherapy, intermediate risk prostate cancer, quality high dose seed implantation, ten‐step method

## Abstract

Dose escalation is key for improved outcomes in intermediate‐risk prostate cancer, including unfavorable intermediate‐risk (UIR) cases. This educational report is designed to provide information about our quality high‐dose ^125^I seed implantation monotherapy technique in which a biologically effective dose (BED) ≧ 200 Gy is applied for treatment of intermediate‐risk prostate cancer. This protocol is named the “Ten‐step Method,” where the rationale and principle of the method are based on the following four goals: (1) The entire prostate should be covered by the prescription isodose distribution with a sufficient margin from the prostatic capsule, achieving high D90 and V100 values by ^125^I seed implantation. (2) The high‐dose cloud (240 Gy) should not invade the urethra or rectum. (3) In order to achieve goals (1) and (2), make the high‐dose cloud intentionally along the periphery (bilateral wall to anterior wall) away from the urethra and rectum. (4) In order to achieve goal (3), seeds at the periphery, except those anterior to the rectal wall, should be placed just 1mm inside the capsule. The data obtained from a total of 137 patients with intermediate‐risk prostate cancer treated with low‐dose‐rate (LDR) monotherapy are shown. The dosimetry parameters were monitored at 1 month after seed implantation by using CT and MRI fusion guidance. The data at 1 month after LDR were: Average D90, BED, and V100 of ^125^I LDR monotherapy were 194.1 Gy, 207.3 Gy, and 99%, respectively. This ten‐step method was reproducible in 137 patients with intermediate‐risk prostate cancer, allowing administration of high‐dose monotherapy with excellent clinical outcomes.

## 1 INTRODUCTION

For an effective radiotherapy in high‐ or intermediate‐risk prostate cancer, a good local control by dose escalation is crucial.^1^, ^2^ Modern prostate brachytherapy provides the advantage of safely delivering a high biologically effective dose (BED) to the prostate to achieve good local control. High‐radiation‐dose seed implantation using a real‐time, intraoperative technique was originally proposed by the group at Mount Sinai School of Medicine.^1^, ^3^

In intermediate‐risk patients, Stone et al., reported that based on three BED dose groups by seed implantation alone, <140, 140–200, and > 200 Gy, biochemical failure‐free survival (BFFS) at 10 years was 52.9%, 74.1%, and 94.3%, respectively.^4^, ^5^

We have recently shown excellent clinical outcomes with low‐dose‐rate (LDR) ^125^I based radiotherapy for intermediate‐risk patients, including a substantial number of unfavorable intermediate‐risk (UIR) cases: BFFS rate of 99.1% at 7 years.^6^ In that study, we concluded that LDR ^125^I brachytherapy alone with a BED of ≧200 Gy is an effective treatment for intermediate‐risk prostate cancer, including UIR cases, thus supporting the above‐mentioned data from the Mount Sinai group.^4^, ^5^ Our clinical outcomes for intermediate‐risk prostate cancer patients have been achieved based on our high‐dose brachytherapy technique. For brachytherapists, including physicists, LDR alone with a BED of **≧**200 Gy requires a highly skilled brachytherapy technique to safely deliver a high radiation dose. In the previous publication,^6^ we declared that the detailed method of the high‐dose implantation technique would be presented separately. Thus, in the present technical report, our LDR ^125^I implant monotherapy method with a BED of 200 Gy is described in detail. The current study aims to disseminate our high‐dose brachytherapy technique by providing implementation specifics.

## 2 MATERIALS AND METHODS

### 2.A ^125^I seed implantation

This LDR brachytherapy implantation method with ^125^I seeds is based on the Mount Sinai real‐time implantation technique (MS method).^1^, ^3^

^125^I seeds (Oncoseed; Nihon Mediphysics Co., Tokyo, Japan) were implanted using a Mick applicator (Mick Radio‐Nuclear Instruments, Inc., Mount Vernon, NY). Planning was performed with the VariSeed 8.0 planning system (Varian Medical Systems, CA, USA). The dosimetry was continually updated, so the plan evolved dynamically as seeds were implanted.

An advantage of the Mick applicator was that the number of seeds per needle could be changed in real time. Therefore, our implantation technique cannot be conducted with stranded seeds or preloaded needles. Two weeks before the seed implantation, we conducted prostatic volume studies on each patient using an ultrasound probe. According to the prostatic volume, we determined the number of seeds to be ordered based on the following nomogram formula.

Nomogram formula: A (the number of seeds) × B (seed activity by air‐kerma strength) × 0.787 = 9.365 + 0.595 × C (prostatic volume: cm^3^).

The number of ordered seeds: 1.15 × A. Seed activity (B): Mostly 0.361 (U), prostate volume≧45 cm^3^ 0.429 (U), prostate volume≧50 cm^3^ 0.502 (U).

Furthermore, we never used the hydrogel spacer because our method included a vigorous process to avoid an overdose to the rectum. The average time for seed implantation is 80 (range 60–100) minutes. The implanted seeds are described in the legend to Table 1, including the number of seeds used and air‐kerma strength.

**Table 1:** Dosimetric parameters of ^125^I seed implantation using the "Ten‐step method” at 1 month and calculated BED in 137 cases

Number of seed LDR monotherapy	n = 137	
Variables	Mean (range) at seed implantation	Mean (range) at one month after seed implanation
Prostate D90 (Gy)	196.2 (173–207.5)	194.1 (156.9–223.8)
BED (Gy)	209.7 (183.5–222.6)	207.3 (165.5–241.4)
V100 (%)	99.8 (98.1–100)	99.0 (94.1–100)
UD30 (Gy)	201.3 (175.1–224.2)	211.7 (155.7–263.2)
R100 (cc)	0.23 (0–1.07)	0.47 (0–2.03)

Average prostate volume (range) of 137 cases is 32.9 (15‐77.0) cm3. D90: minimal dose (Gy) received by 90% of the prostate. BED: biologically effective dose. V100: The percentage prostate volume receiving 100% of the prescribed minimal peripheral dose; UD30: Minimal dose (Gy) received by 30% of the urethra; R100: Rectal volume (cm^3^ ) receiving 100% of the prescribed dose. Average (range) of air‐kerma strength of the implanted seeds is 0.390 (U): range 0.361–0.501 (U). Average number (range) of implanted seeds is 102 (75–144).

The prescription dose of seed implantation was set at 144 Gy for LDR ^125^I monotherapy: we set D90 at 190 Gy. For this method, we commonly used ^125^I seed activity at 0.361 (U) as described in our previous report.^6^

This method was conducted in compliance with AAPM recommendations (Report of Task Group 137) on dose description, reporting methods for LDR brachytherapy for prostate cancer including real‐time implantation method, OAR monitoring, timing, and method of the postimplantation dosimetry.^7^

The equation of BED: BED = ($\left(R0/\lambda \right)\left\{1+\left[R0/\left(\mu +\lambda \right)\left(\alpha /\beta \right)\right]\right\}\;=\;\text{D9}0\left[1+\text{D9}0\times \left(0.693/\text{T1}/2\right)\times \left(0.693/\text{t1}/2+0.693/\text{T1}/2\right)‐1\times \left(\alpha /\beta \right)‐1\right]$.

R_0_ = initial dose rate of implant = (D90)(λ); λ= radioactive decay constant = 0.693/T_1/2_; T_1/2_ = radioactive half‐life of isotope; μ= repair rate constant = 0.693/t_1/2_; and t_1/2_ = tissue repair half‐time. The specific values used for these constants for prostate carcinoma were α/β = 2 Gy, t_1/2_ = 1 h, T_1/2 =_ 60 days for I‐125.^8^, ^9^, ^10^

By convention, the unit of BED calculated when α/β = 2 Gy is Gy_2_. AAPM recommendations used α/β = 3 Gy while the author and others previous reports^8^, ^9^, ^10^ used α/β = 2 Gy in accordance with the above‐mentioned formula.

Therefore, by using α/β = 2 Gy, the standard dose and BED of^125^ I seed implantation according to the report of Task Group 137 were 145 Gy and 152.4 Gy, respectively, while our standard dose and BED are 190 Gy and 202.7 Gy, respectively.

### 2.B Ten‐step method

The method is composed of ten steps, beginning with the positioning of the ultrasound probe and ending with the last seed placement at the midline of the apex between the urethra and rectum. The final step is a confirmation of the dose cloud and dosimetry.

The ten‐step method is based on the following rationale. First of all, placing a sufficient number of seeds in the peripheral region away from the urethra and rectum. This insures a good treatment margin beyond the prostate capsule. The rationale and principle of this method achieve the following four goals:

(1) The entire prostate should be covered by the prescription dose cloud with a sufficient margin (5–7 mm) from the capsule in all directions except for those anterior to the rectal wall, thus achieving high D90 (D90 > 190 Gy) and V100 (V100 > 99%) values by ^125^I seed implantation.
(2) A high‐dose cloud (240 Gy) should not approach the urethra or rectum.
(3) In order to achieve goals (1) and (2), it is necessary to make the high‐dose cloud intentionally along the periphery (bilateral wall to anterior wall) away from the urethra and rectum.
(4) In order to achieve goal (3), seeds at the periphery, except those anterior to the rectal wall, should be placed just 1mm inside the capsule.

Each of the ten steps of the method is described below:

① Positioning and fixation of ultrasound probe.

This is the first and the most crucial step of this method to conduct quality seed implantation.

**Fig. 1:**
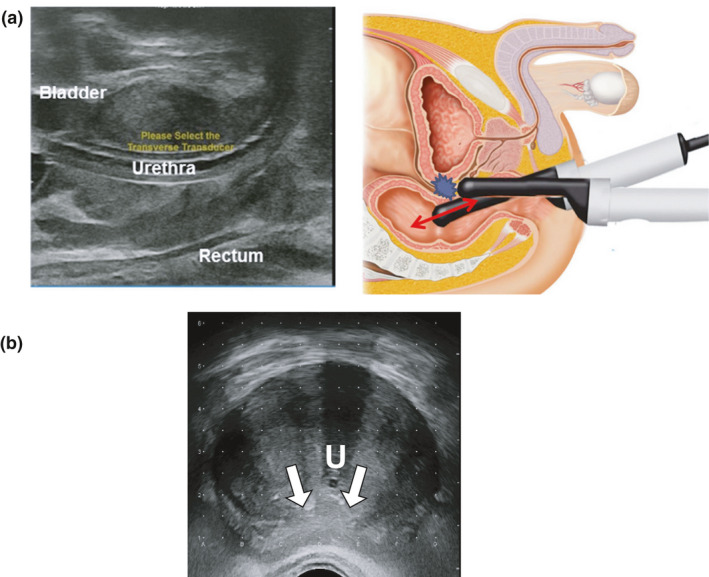
Positioning and fixation of ultrasound probe. (a) Step ①‐1, by a sagittal image, the ultrasound probe angle should be set so the advancement of the probe proceeds smoothly from the apex to the base without colliding the probe against the anterior rectal wall. (b) Step ①‐2, at the largest transverse image section, the ultrasound probe should be fixed, so the distance between the needle holes that are the most anterior to the rectal wall (c‐1.5 and d‐1.5) and the most posterior surface of the prostate is kept at 5–7 mm (white arrows). The urethral position (U) need not be necessarily in the center of the transverse image.

【 Step ① −1 】By a sagittal image, the ultrasound probe angle should be set so the advancement of the probe proceeds smoothly from the apex to the base without colliding the probe against the anterior rectal wall [Fig. 1(a)].

【 Step ① −2 】

Then, at the largest transverse image section, the ultrasound probe should be fixed so the distance between the needle holes that are the most anterior to the rectal wall (c‐1.5 and d‐1.5) and the most posterior surface of the prostate is kept at 5–7 mm [Fig. 1(b)].

① Acquisition of transverse images and fine adjustment of the target volume by a sagittal image.

**Fig. 2:**
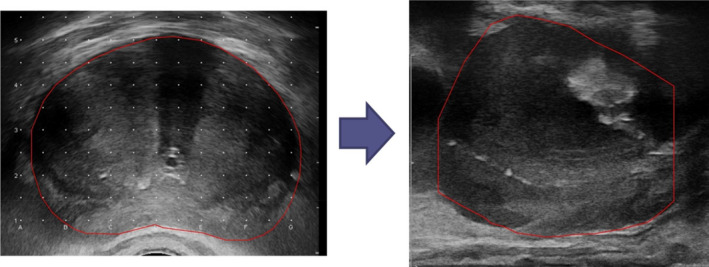
Acquisition of transverse images and fine adjustment of the target volume by a sagittal image. Step ②, acquisition and contouring of the transverse images of the prostate from the base to the apex is done (Left). Next, a fine adjustment of the target volume is conducted by overlaying the target volume onto the sagittal image of the prostate. This step allows us to produce an accurate target volume (red line) at the base and apex. (Right).

【 Step ② 】First, acquisition and contouring of the transverse images of the prostate from the base to the apex is done. Then, a fine adjustment of the target volume is conducted by overlaying the target volume onto the sagittal image of the prostate. This allows us to produce an accurate target volume at the base and apex [Fig. 2].

② Peripheral needle placement and readjustment of the target volume.

**Fig. 3:**
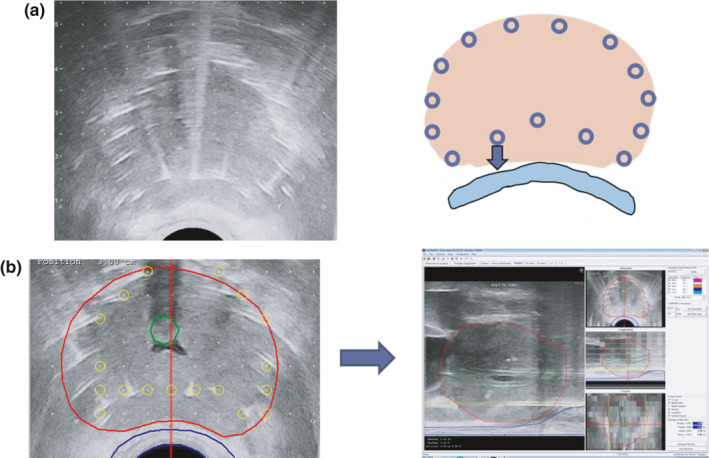
Placement of peripheral needles and readjustment of the target volume. (a) Step ③‐1, at this step, peripheral (bilateral, anterior, and posterior) needles, including the needles anterior to the rectum, are placed. Lateral and anterior needles should be placed just inside the prostatic capsule. The distance to the nearby needles should be 5‐7mm (not more than 7mm). Needles should also be placed at the bottom of bilateral corners (right and left). As described in Step ①, the distance between the needles above the rectum (c‐1.5 and d‐1.5) and those at the posterior surface of the prostate should be kept at 5–7 mm: care must be taken “not to get too close to the posterior surface of the prostate in order to avoid overdosing the anterior rectal wall (blue arrow). (b) Step ③‐2, after completing insertion of the peripheral needles, reacquisition of the transverse images should be conducted from the base to the apex. Then, the final target volume (red line) is determined by the alignment with the sagittal image.

【 Step ③ −1 】At this step, peripheral (bilateral, anterior, and posterior) needles, including the needles anterior to the rectum, are inserted [Fig. 3(a)]. Lateral and anterior needles should be placed just inside the prostatic capsule. The distance to the nearby needles should be 5–7 mm. There should be no restriction on the number of peripheral needles [Fig. 3(a)]. As shown in Step ①, the distance between the needles above the rectum (c‐1.5 and d‐1.5) and those at the posterior surface of the prostate should be kept at 5–7 mm.

During the needle insertion, the operator must take care to not allow significant prostate motion by needle placement. In this regard, appropriate positioning and fixation of the ultrasound probe in step ① is crucial and key to succes**s** for this method.

【 Step ③ −2 】After completing insertion of the peripheral needles, reacquisition of the transverse images should be conducted from the base to the apex. Only minor adjustment of the target volume is usually required at this stage as long as the positioning and fixation of the ultrasound probe by the operator (step ①) was appropriate [Fig. 3(b)]. Then, the final target volume is determined by the alignment with the sagittal image.

③ Positioning of the peripheral needles and creation of putative planning.

**Fig. 4:**
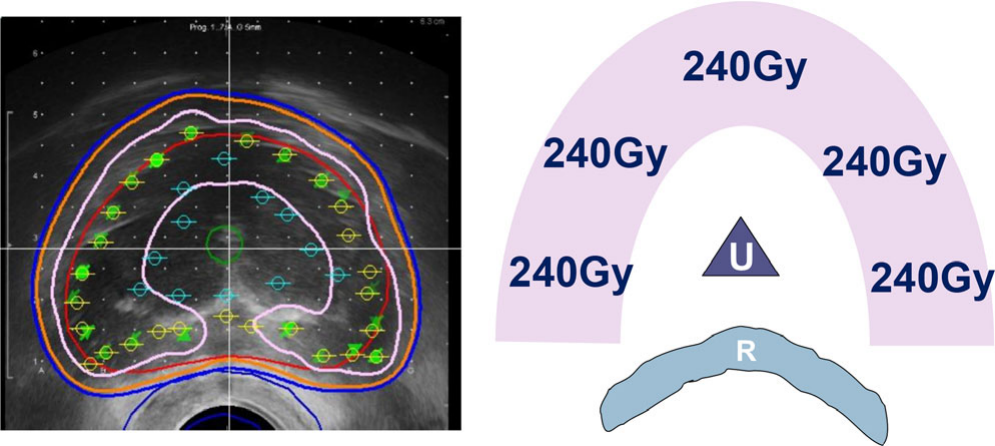
Positioning of the peripheral needles and creation of putative planning. Step ④‐1, the accurate positioning of each peripheral needle (green circles) is conducted at the largest transverse image of the prostate (Left). Step ④‐2, next, the positions of the interior needles (blue circles) are assigned (Left). The positions of these needles should be 7–10 mm away from the urethra at the apex and base. This placement is important in order to avoid overdosing the urethra. Then, putative planning is made (red line: target prostate; orange line: 160 Gy; blue line prescription dose 144 Gy; pink line: 240 Gy). At this stage, we try to make a high‐dose band (240 Gy, pink) intentionally along the bilateral corners and the lateral and anterior walls at the largest transverse prostate image. The rationale for this step is making a high‐dose band (pink area) at peripheral areas at a sufficient distance from the urethra (U) and rectal wall (R) (Right).

【 Step ④ −1 】The accurate positioning of each peripheral needle is conducted at the largest transverse image of the prostate [Fig. 4 Left].

【 Step ④ −2 】 Next, the positions of the interior needles (blue circles) are assigned [Fig. 4 Left]. The positions of these needles should be 7–10mm away from the urethra at the apex and base. This placement is important in order to avoid overdosing the urethra. [Fig. 4]. Then, additional treatment planning is performed, making a high‐dose band (240 Gy) **i**ntentionally along the bilateral corners and the lateral and anterior walls at the largest transverse prostate image [Fig. 4 Right].

⑤ Seed implantation through the peripheral needles.

**Fig. 5:**
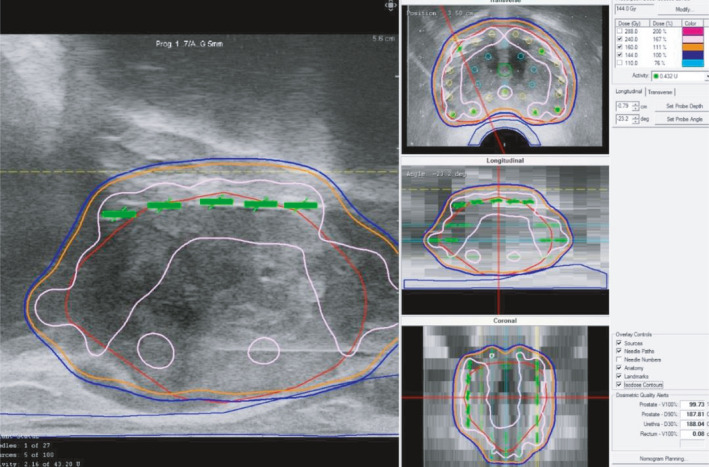
Seed implantation through the peripheral needles. The principle for seed deposition through the peripheral needles is as follows: this figure illustrates the peripheral needle at 10 o’clock, which is far away from both urethra and rectum. In each peripheral needle, a sufficient number of seeds should be deposited so that not only a 160 Gy area (orange line) but also a high‐dose area (240 Gy, pink line) extends over the entire target volume (red), and a 160 Gy area (orange) covers the target volume with a good margin (5–10 mm) at the level of each peripheral needle: Build a high‐dose band at the peripheral area at a distance from the urethra and rectal wall. The number of seeds on each needle should be increased or reduced in order to meet the above‐mentioned requirements based on the real‐time isodose cloud of each needle.

【 Step ⑤】The principle for seed implantation through the peripheral needles is as follows: In each peripheral needle, a sufficient number of seeds should be deposited so that a high‐dose (240 Gy) area extends over the entire target volume in a sagittal image (at the prostate area away from the urethra), and a 160 Gy area covers the target volume with a good margin (5–10 mm) at the level of each peripheral needle [Fig. 5]: Build a high‐dose band at the peripheral area at a sufficient distance (at least 10 mm) from the urethra and at least 5 mm from the rectal wall [Figs. 4 and 5].

Importantly, the number of seeds on each needle should be increased or reduced in order to meet the above‐mentioned requirements based on the real‐time iso‐dose distribution of each needle [Figs. 4 and 5]. We should remember that a high‐dose area can be made intentionally in the peripheral zone over the capsule without overdosing the urethra and rectum. This will lead to a high‐quality implant with high D90 and V100 values. Therefore, the number of implanted seeds in this area should be carefully adjusted in real‐time, and preloaded needles would not suffice.

⑥ Interior needle placement: confirmation of distance between urethra and interior needles at the base and apex.

Note that Steps ⑥ and ⑦ are critical steps to control the urethral dose.

**Fig. 6:**
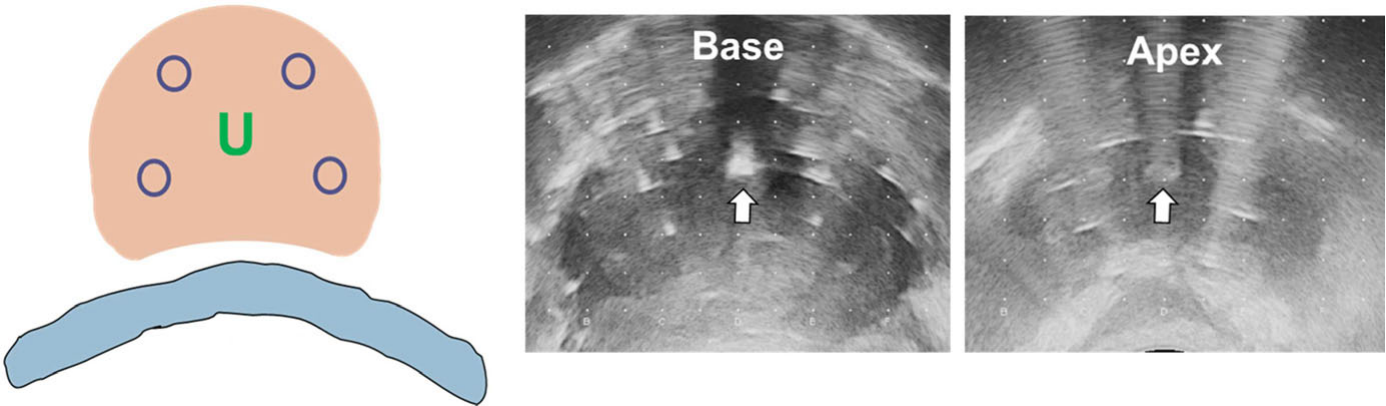
Interior needle placement: confirmation of the distance between urethra and interior needles at base and apex. Step ⑥, after insertion of interior needles, the urethral catheter is removed. In removing the catheter, bubbled lubricant jelly was left in the urethral space so the natural appearance of the urethra is visualized. Then, the distance between the interior needles and the urethra at the base and apex is confirmed. If the distance is short (less than 7 mm), use one grid outer needle hole at either base or apex. See the distance between the urethra with bubbled lubricant jelly (white arrow) and the position of the interior needles at the base and apex.

【 Step ⑥ 】After insertion of interior needles, the urethral catheter is removed. In removing the catheter, bubbled lubricant jelly was left in the urethral space so that the natural appearance of the urethra is visualized. Then the distance between the interior needles and the urethra at the base and apex is confirmed. If the distance is short (less than 5 mm), use one grid outer needle hole at either base or apex [Fig. 6].

⑦ Seed deposition from interior needles after confirmation of urethral dose at the base and apex.

**Fig. 7:**
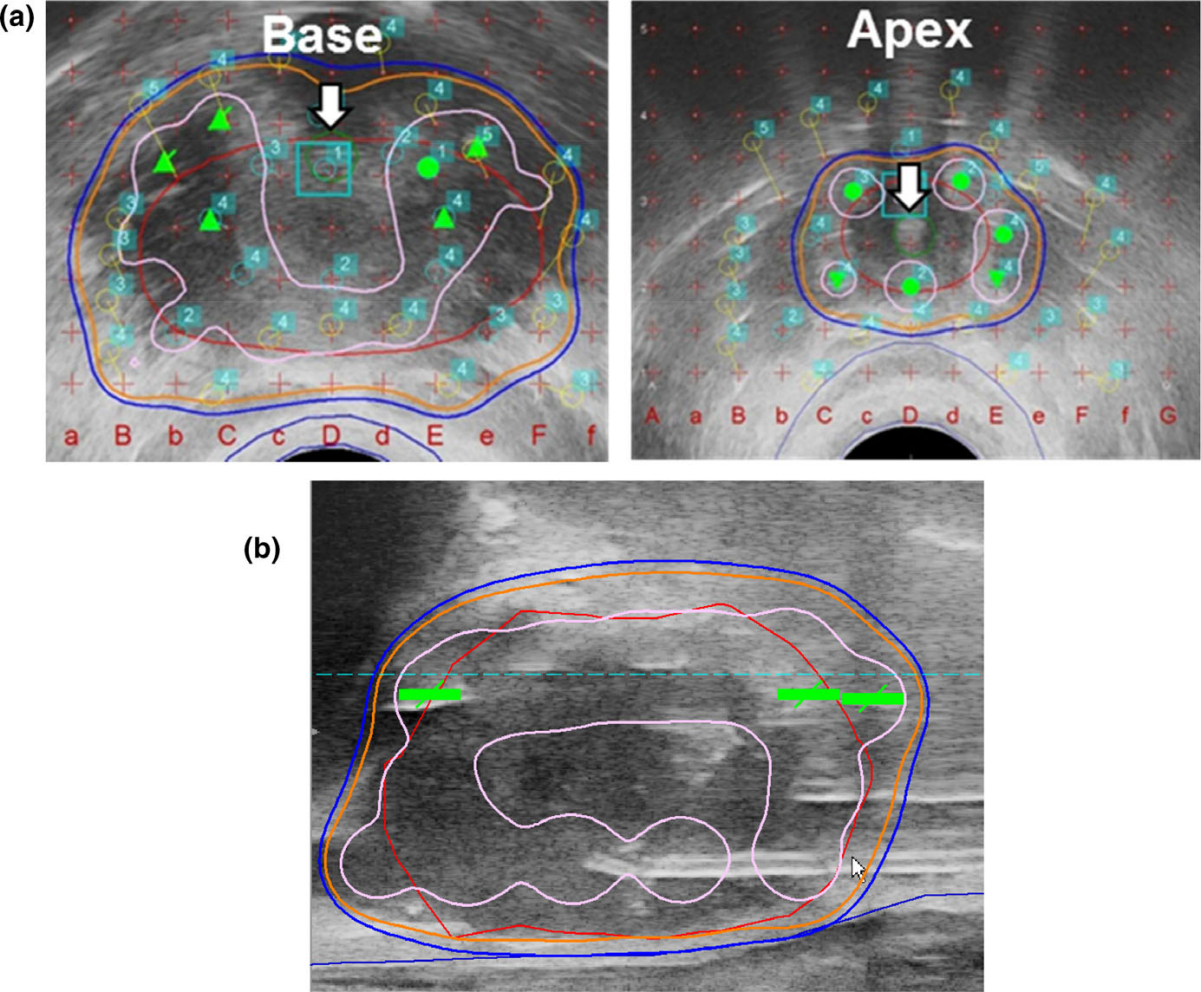
Seed deposition from interior needles after confirmation of urethral dose at the base and apex. (a) Step ⑦‐1, before placing seeds from interior needles, the isodose distribution at the base and apex should be confirmed, so the high‐dose area (240 Gy, pink line) does not approach the urethra (white arrow), and the target volume (red line) is sufficiently covered with a dose cloud of 160 Gy (orange line) with a good margin (5–10 mm). (b) ⑦‐2, for seed deposition from interior needles, all seeds should be placed only at either base or apex: Seeds should be deposited so the dose area of 160 Gy (orange line) covers the prostate target (red line) with a good margin (5–10 mm).

【 Step ⑦ −1 】 Before placing seeds from interior needles, the isodose distribution at the base and apex should be confirmed so that the high‐dose area (240 Gy) does not approach the urethra, and the target volume is sufficiently covered with a dose cloud of 160 Gy with a good margin (5–10 mm) [Fig. 7].

We routinely use one or two D line needles as the interior needles above the urethra.

As long as there is real‐time monitoring with axial and sagittal ultrasound images to avoid seed placement within 7 mm of the urethra, the D column can be safely utilized. Furthermore, use of the D line needle can contribute to high D90 and V100 values by avoiding overdose to the urethra.

【 Step ⑦ −2 】For seed deposition from interior needles, all seeds should be placed only at either base or apex: Seeds should be deposited so that the dose area of 160 Gy covers the entire prostate with a good margin [Fig. 7]. We often deposit two seeds at the apex with a distance of 10–12 mm from the urethra [Fig. 7]. This technique allows good dose coverage at the apex by avoiding overdosing the urethra [Fig. 7(b)].

⑧ Adjustment of posterior needle position above the rectum.

**Fig. 8:**
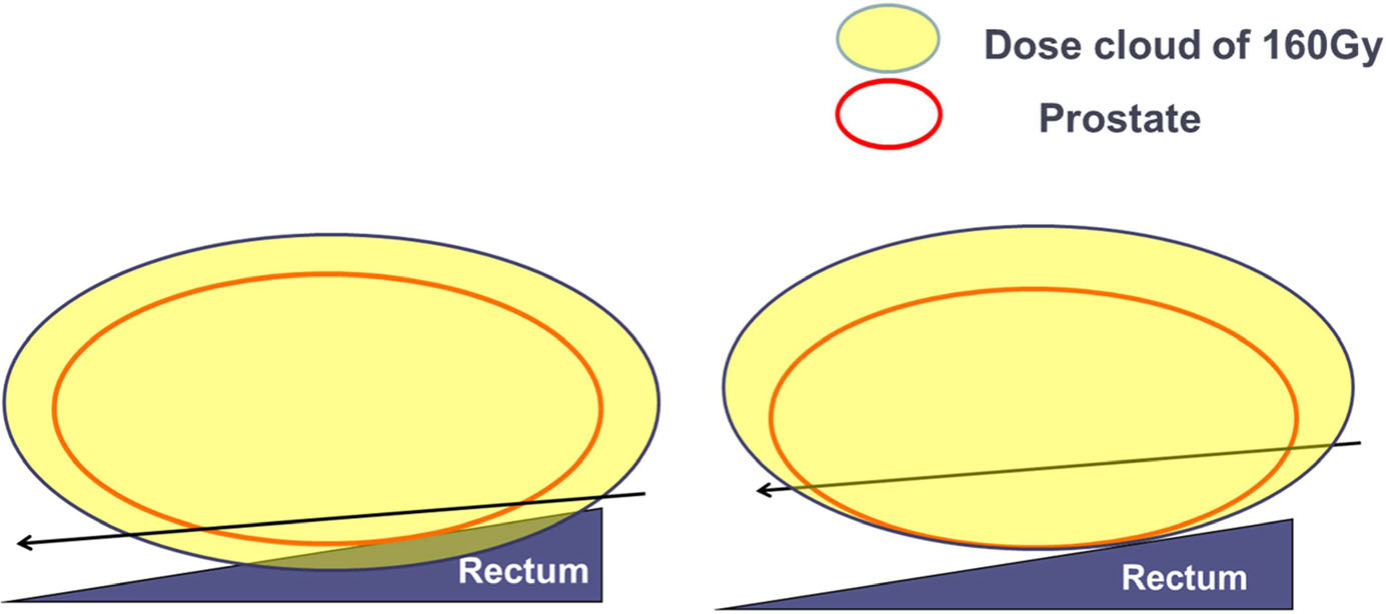
Adjustment of posterior needle position above the rectum. Step ⑧, at this stage, only three to five needles above the rectum remain to be implanted. If any needle is too close to the rectum, and the dose cloud of 160 Gy could invade the rectum (Left), the needle should be reinserted and shifted into the upper position away from the rectal wall in order to reduce the rectal dose (Right).

【 Step ⑧ 】 At this stage, only three to five needles above the rectum remain to be implanted. If any needle is located too close to the rectum, and the dose cloud of 160 Gy could invade the rectum, the needle should be reinserted and shifted into the upper position away from the rectal wall in order to reduce the rectal dose. Then, the distance between these needles and the posterior surface of the prostatic capsule should be kept at 5‐7 mm [Fig. 8].

⑨ Seed implantation from the needles above the rectum.

**Fig. 9:**
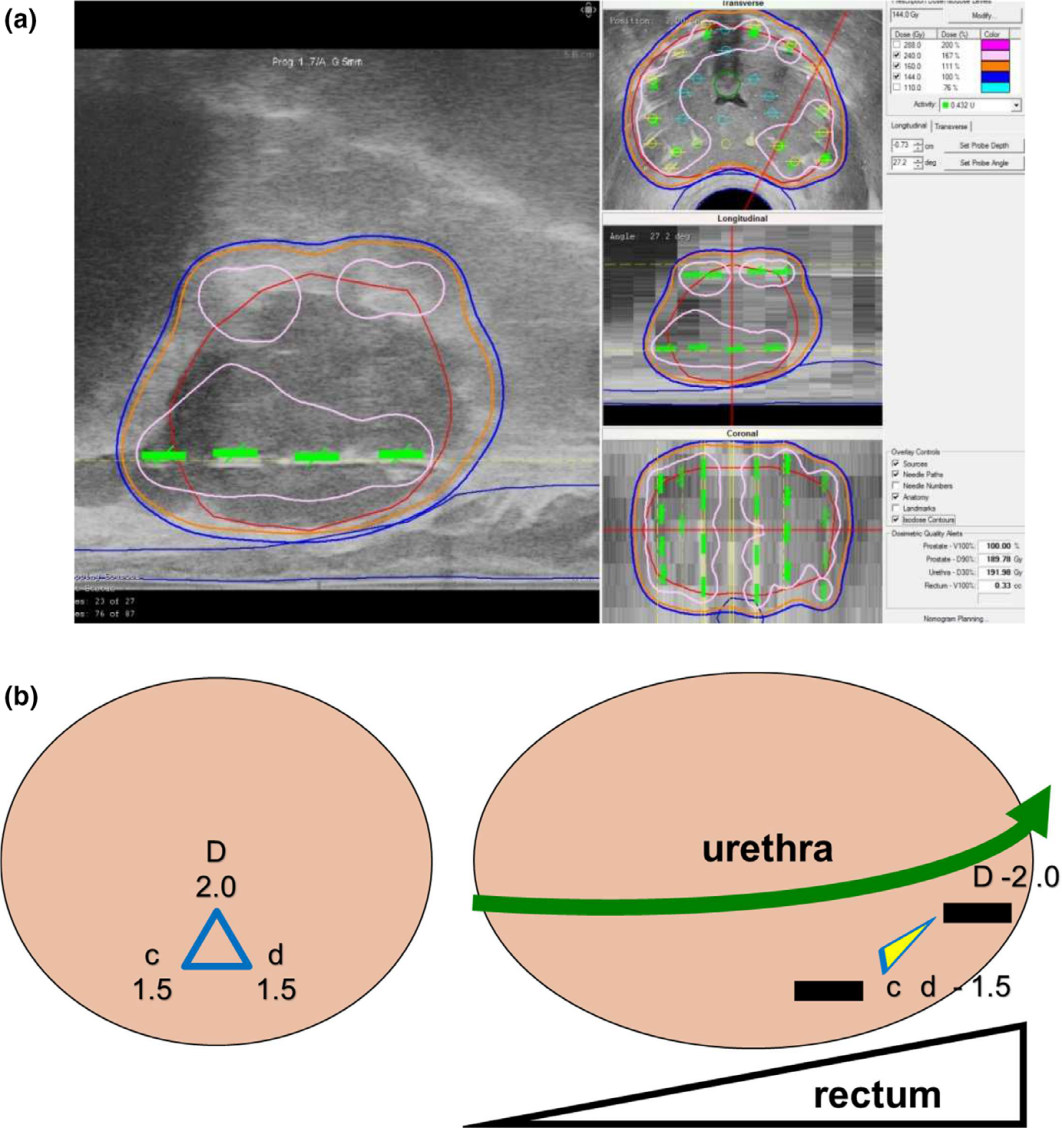
Seed deposition from the needles above the rectum. (a) Step ⑨‐1, after adjustment of the needle positions above the rectum, seed insertions at 1.5 of c and d columns are done. At 1.5 of c and d columns, the seeds closest to the apex should be deposited inside the prostate a few millimeters from the apex in order to reduce the rectal dose. (b) Step ⑨‐2, the final seed between the urethra and rectum (usually D‐2.0) is deposited. Before this final seed deposition, the positions of the deposited seeds near the apex (c, d‐1.5) should be visualized and confirmed, so that these three seeds should form a three‐dimensional triangle. Note that these three seeds should not be placed in line in order to avoid creating a hot spot at the anterior rectal wall at the apex.

【 Step ⑨ −1 】 After adjustment of the needle positions above the rectum, seed insertions at 1.5 of c and d columns are done. At 1.5 of c and d columns, the seeds closest to the apex should be deposited a few millimeters inside the prostate in order to reduce the rectal dose [Fig. 9].

【 Step ⑨ −2 】 Finally, the final seed between the urethra and rectum (usually D‐2.0) is deposited. In this step, the positions of the already deposited seeds near the apex (c, d‐1.5) should be visualized by ultrasound. These three seeds should form a three‐dimensional triangle. Note that these three seeds should not be placed in line in order to avoid creating a hot spot at the anterior rectal wall at the apex [Fig. 9(b)].

⑩ Final step: confirmation of dosimetry.

**Fig. 10:**
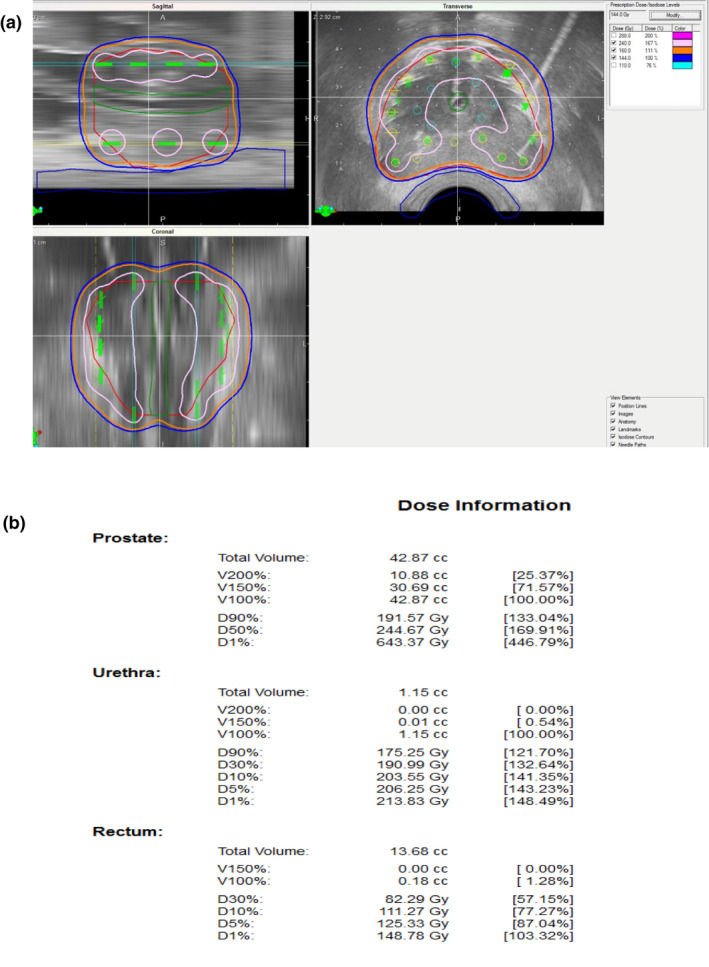
Final step: confirmation of dosimetry. (a): As the final step, check the entire dose distribution for all 2D views and confirm that the entire prostate target is covered with 160 Gy (orange). Note that the high‐dose band (240 Gy, pink) does not invade the urethra and rectum C (b): Confirm parameters including OAR.

【 Step ⑩ 】As the final step, we check the entire dose distribution for all 2D views (sagittal, transverse, coronal) and confirm that the entire prostate target is covered with 160 Gy [Fig. 10(a)].

Note that the high‐dose cloud (240 Gy) does not invade the urethra and rectum.

D90 should be over 190 Gy and V100 should be over 99%. Upon OAR parameters, UD30 should be under 215 Gy and R100 should be under 0.5cm^3^ [Fig. 10(b)].

### 2.C Patients

This study has been approved and monitored by our institutional ethics committee. Between 2014 and 2016, a total of 137 patients with intermediate‐risk prostate cancer treated by LDR ^125^I mono therapy using the ten‐step method were analyzed. All of the 137 patients visited the author’s specialized outpatient clinic for prostate brachytherapy to seek the author’s medical advice and treatment.

Then prostate biopsy samples obtained in other hospitals from nationwide were re‐examined by an experienced pathologist, who is an expert in diagnosis of prostate cancer. Based on the pathological confirmation with MRI and physical findings, the 137 patients were diagnosed as having intermediate‐risk prostate cancer. The highest PSA values before the seed implantation were recorded: The average PSA was 8.6ng/ml (range 3.12‐20ng/ml). Intermediate‐risk prostate cancer divided into “favorable” and “unfavorable” subgroups was used. Favorable intermediate‐risk (FIR): one intermediate‐risk factor with Gleason score 3 + 4. Unfavorable intermediate‐risk (UIR): Patients defined as unfavorable intermediate risk if Gleason 4 + 3 = 7 or > 1 intermediate‐risk factors (cT2b, c, PSA 10–20, Gleason 3 + 4 = 7). In the present series, 49 cases (36%) were FIR cases and 88 cases (64%) were UIR cases. Only four cases (2.9%) received short‐term hormonal therapy by other hospitals. Hormonal therapy was suspended after consultation in our department.

### 2.D Evaluation of Postimplantation dosimetry

Postimplant dosimetry was carried out at 1 month after seed implantation by using CT and MRI fusion guidance.^6^, ^11^ The biologically effective dose (BED) was calculated using an α/β ratio of 2.^9^

### 2.E Toxicity

Acute toxicity was defined when symptoms developed within 6 months after implantation. Late toxicity was defined when any kind of symptom developed after 6 months or occurred within the first 6 months and persisted for more than 1 year. Toxicity was recorded by the Common Terminology Criteria for Adverse Events version 4.0.

## 3 RESULTS

### 3.1 A. Dosimetric parameters and BED

The dosimetric parameters at seed implantation and 1 month after seed implantation including total BED of 137 patients are shown in Table 1. Of those, the prostate volume was over 40 cm^3^ in 27 cases. The dosimetric parameters at seed implantation and 1 month after seed implantation including total BED of the 27 patients are shown in Table 2.

**Table 2:** Dosimetric parameters of ^125^I seed implantation using the "Ten‐step method” at 1 month and calculated BED in 27 cases with prostate volumes over 40 **cm^3^** is shown

Number of seed LDR monotherapy	n = 27	
Variables	Mean (range) at seed implantation	Mean (range) at one month after seed implanation
Prostate D90 (Gy)	194.5 (173.0–199.0)	192.9 (167.4–223.8)
BED (Gy)	207.7 (183.5–213.1)	205.9 (177.2–241.4)
V100 (%)	99.7 (98.1–100)	99.3 (97.1–100)
UD30 (Gy)	197.6 (175.1–212.8)	205.8 (155.7–259.2)
R100 (cc)	0.321 (0–1.07)	0.68 (0–2.03)

Average prostate volume (range) of 27 cases is 46.6 (40‐77.0) cm3. D90: minimal dose (Gy) received by 90% of the prostate. BED: biologically effective dose. V100: The percentage prostate volume receiving 100% of the prescribed minimal peripheral dose; UD30: Minimal dose (Gy) received by 30% of the urethra; R100: Rectal volume (cm^3^) receiving 100% of the prescribed dose.

### 3.2 B. Toxicity

Acute grade 2 genitourinary (GU) and gastrointestinal (GI) toxicity was experienced by 21 patients (15%) and one patient (0.7%), respectively. Late grade 2 genitourinary (GU) and gastrointestinal (GI) toxicity was experienced by nine patients (7%) and one patient (0.7%), respectively. No Grade 3 or higher toxicities were observed in the present series. None of the patients experienced urethral stricture, TUR‐P (transurethral resection of prostate), or recto‐urethral fistula. Acute urinary retention requiring temporary urethral catheterization for 1 or 2 weeks was observed in two patients (1.4%).

### 3.C Efficacy of the treatment

Mid‐term efficacy of the treatment by this ten‐step method has already been published in our previous study as a part of clinical outcomes for intermediate‐risk prostate cancer patients^6^: BFFS rate at 7 years by LDR alone or with a BED of 200 Gy was 99%. Notably, none of the patients has experienced local recurrence so far.

## 4 DISCUSSION

Radical prostatectomy is one of the standard treatment modalities for intermediate‐risk prostate cancer. Reese et al. reported BFFS in a cohort of 4,164 intermediate‐risk patients treated by radical prostatectomy.^12^ The results showed that the 5‐year BFFS differs significantly between FIR patients and UIR patients (83% and 64.3%, respectively).^12^ Similarly, a difference in BFFS between FIR patients and UIR patients was reported in cases treated by external beam radiotherapy (EBRT) of 81 Gy.^13^

In their report, the estimated 8‐year BFFS are 86.1% and 71.1% for FIR patients and UIR patients, respectively. Apart from the issue whether these outcomes by radical prostatectomy or EBRT are satisfactory or not, considering the patients' quality of life, physicians involved in prostate cancer treatment should have serious concerns about the fact that failure of the initial treatment imposes many kinds of physical, psychological, and economical burdens to the patients, such as toxicities caused by salvage hormonal therapy or radiotherapy, anxiety about developing castration‐resistant prostate cancer (CRPC), and financial insecurity after second or third salvage drug therapy, which is very expensive. Furthermore, the reader is reminded of the medical costs for prostate cancer treatment. Failure of the initial treatment causes a serious health insurance cost. The above‐mentioned problems could even diminish the benefit of PSA screening itself, if the problems remain unresolved. It can be argued that good clinical outcomes are achieved with contemporary doses of 145 Gy by ^125^I LDR monotherapy: The data from 11 Italian institutions showed the clinical outcomes of 2,237 cases (the largest European LDR monotherapy study) of low‐ and intermediate‐risk prostate cancer treated by ^125^I LDR monotherapy of D90** = **145 Gy.^14^ The result showed BFFS rates at 7 years of 92.8% and 78.4%, in low‐ and intermediate‐risk patients, respectively.

Thus, contemporary doses of 145 Gy by ^125^I LDR monotherapy may possibly be justified only in low‐risk prostate cancer patients who are also good candidates for active surveillance.

As a brachytherapist with extensive experience, the author has published good clinical outcomes by dose escalation in high‐risk and very high‐risk prostate cancers, including regional lymph node metastasis: A BFFS rate of 95.2 % at 5 years by LDR in combination with EBRT of BED > 220 Gy.^11^ In intermediate‐risk cancer patients, it has been debated whether there is a need for supplemental EBRT when applying LDR‐based radiotherapy in intermediate‐risk cancer patients.^5^, ^15^ However, in those debates, there is a clear agreement that dose escalation is the key to improve outcomes for intermediate‐risk patients, including UIR patients.^5^, ^15^ Stone et al. reported that the patients with intermediate‐risk prostate cancer treated by seed implantation alone in three BED dose groups (α/β ratio of 2) of < 140, 140–200, and > 200 Gy, the BFFS at 10 years was 52.9%, 74.1%, and 94.3% in intermediate‐risk prostate cancer, respectively.^4^, ^5^ According to the previous reports of clinical outcome on patients with intermediate‐risk prostate cancer from high‐volume brachytherapy centers, standard BED or D90 by LDR alone were not high enough, based on the data by Stone et al.^4^, ^5^: Zelefsky et al., reported BFFS of 89% at 5 years with a median D90 of 173 Gy and median BED (α/β ratio of 2) of 183.5Gy.^16^ Similarly, Kimura et al., reported BFFS of 79.4% at 8 years with a median D90 of 163.7 Gy and a median BED (α/β ratio of 2) of 178.4 Gy.^17^

Indeed, the Group at Mount Sinai School of Medicine has recommended a BED (α/β ratio of 2) of 200 Gy by LDR alone for intermediate‐risk prostate cancer patients^1^, ^3^: LDR ^125^I monotherapy using a minimum D90 implant of 180 Gy or greater has been recommend for intermediate‐risk prostate cancer patients.^18^

We have recently shown excellent clinical outcomes with ^125^I LDR‐based radiotherapy in intermediate‐risk patients including a significant number of unfavorable intermediate‐risk (UIR) cases: BFFS of 99.1% at 7 years.^6^ In the report, we have concluded that by technical evolution, ^125^I LDR alone with a BED of 200 Gy is an optimal treatment for intermediate‐risk prostate cancer patients, including UIR cases; This is in good agreement with the data by the group of Mount Sinai School of Medicine.^1^, ^4^, ^5^ However, for brachytherapists or physicists, LDR alone with a BED (α/β ratio of 2) of 200 Gy requires a highly skilled technique to deliver a high radiation dose safely. For example, in the report by Kao et al., the median D90 was 197 Gy, median rectal V100 was 1.00 cm^3^ (range 0–6.19 cm^3^) with a prescription dose of 160 Gy ^125^I LDR monotherapy. Acute urinary retention was observed in 10.7%.^18^ On the contrary, in our series, the median D90 was 194.1 Gy, the median rectal V100 was 0.47 cm^3^ (range 0–2.03 cm^3^) with a prescription dose of 144 Gy I^125^ monotherapy. Acute urinary retention was observed only in 1.4%.

The present protocol is an advanced method of real‐time, intraoperative technique by the group at Mount Sinai School of Medicine (MS method). There are a few different points among MS method and ten‐step method. Ten‐step method advocates the founding principles with four goals to achieve quality high‐dose implantation which is not described in MS method. MS method consists of two phases (Real‐Time Brachytherapy (nycprostatecancerexpert.com): The first phase is called the peripheral phase and involves insertion of needles into the largest transverse diameter of the gland approximately 1 cm apart. In ten‐step method, the distance to the nearby peripheral needles should be 5‐7mm (step ④ ). In MS method, approximately 75% of the radioactivity that is implanted is placed within the prostate via peripheral needles. The ratio of the number of implanted seeds among peripheral needles and interior needles is set for 3:1 in MS method. Ten‐step method does not apply such a ratio for the number of implanted seeds between the peripheral and interior needles. MS method determines the number of peripheral needles before implantation by a nomogram based specific formula. In ten‐step method, there is no restriction on the number of peripheral needles. The number of seeds per each peripheral needle is not determined by nomogram, but by the concept that a sufficient number of seeds should be deposited so that a high‐dose (240 Gy) area extends over the entire target volume in a sagittal image (step ⑤). The second phase of MS method involves the placement of interior needles in such a way that the needles cover the apex and base of the gland. The remaining 25% of the activity is implanted through these interior needles.

In the Ten‐step method, there is no 25% rule: the number of seeds per each interior needle is determined by monitoring isodose cloud distribution and the distance between the interior needles and urethra at the base and apex (step ⑦ ).

In MS method, all of the seed implantation via peripheral needles including above the rectum is finished before seed implantation via interior needles.

The author considered that seed implantation via the needles above the rectum should be conducted after seed implantation via interior needles to avoid increasing radiation exposure to the rectal wall: Specifically Ten‐step method incorporated “triangle technique” in the step ⑨ to avoid creating a hot spot at the anterior rectal wall at the apex.

Thus, in order to share our experience with the quality high‐dose seed implantation technique for prostate cancer, this author has presented this detailed technical practice. In addition to high cure rates, the advantage of this method is the cost‐effectiveness for the patients. The patients can omit supplemental EBRT. This can reduce the length of treatment and eliminate the cost of supplemental EBRT. Furthermore, our method does not require hormonal therapy in most cases because the implant quality and dose are constantly high even in cases with large prostates.

A disadvantage of this method is that the average operation time for seed implantation is 80 minutes on average under lumbar anesthesia, which may be longer than with preloaded needles.

This author hopes that this article will help brachytherapists or physicists achieve high‐dose implants consistently and safely.

## 5 CONCLUSION

The Ten‐Step Method of High‐Dose LDR ^125^I Brachytherapy can achieve high‐dose implants (BED≧200 Gy) on a consistent basis. This method enables brachytherapists to achieve high cure rates for intermediate‐risk prostate cancer, including UIR cases, without EBRT and ADT. Application of this method will result in cost‐effectiveness.

## CONFLICT OF INTEREST

Keisei Okamoto solely designed and invented this Ten‐Steps Method: A quality high‐dose ^125^I seed implantation technique for prostate cancer. Brachytherapists and physicists who have interest in this method can freely have direct contact with Keisei Okamoto (keiseiok814@gmail.com).

Keisei Okamoto was associated with the Department of Brachytherapy for Prostate Cancer endowed by Nihon Medi‐Physics Co., Ltd. Part of this method has been introduced and circulated as an Japanese educational leaflet for Japanese brachytherapists in collaboration with Nihon Medi‐Physics Co., Ltd.
